# Assessment of the efficacy of Chinese patent medicine on treating pain caused by prostate cancer: A protocol for systematic review and meta analysis

**DOI:** 10.1097/MD.0000000000017820

**Published:** 2019-12-20

**Authors:** Xiaoyong Gong, Ji-sheng Wang, Xu-dong Yu, Rui-jia Liu, Li-yuan Chu, Yuan-yuan Li, Yi Lei, Hong Li

**Affiliations:** a The Second Affiliated Hospital of Shaanxi University of Chinese Medicine, Xianyang, Xianyang, Shaanxi; b Graduate School of Beijing University of Chinese Medicine; c Department of Andrology; d The First Department of Neurology; e Department of Oncology, Dongzhimen Hospital, Beijing University of Chinese Medicine, Beijing, China.

**Keywords:** Chinese patent medicine, pain, prostate cancer, systematic review

## Abstract

**Introduction:**

With the development of economy and the acceleration of population aging, Prostate cancer (PCa) has presented a situation of high morbidity and mortality worldwide. The recent studies have shown that Chinese patent medicine combined with endocrine therapy in the treatment of prostate cancer not only plays a synergistic role in enhancing the efficacy. This review hopes to adopt meta-analysis to evaluate the efficacy and safety of Chinese patent medicine in the treatment of pain caused by prostate cancer and provides evidence for its application in clinical practice.

**Methods and analysis:**

We will search for PubMed, Cochrane Library, AMED, EMbase, WorldSciNet; Nature, Science online and China Journal Full-text Database (CNKI), China Biomedical Literature CD-ROM Database (CBM), and related randomized controlled trials included in the China Resources Database. The time is limited from the construction of the library to June 2019. We will use the criteria provided by Cochrane 5.1.0 for quality assessment and risk assessment of the included studies, and use the Revman 5.3 and Stata13.0 software for meta-analysis of the effectiveness, recurrence rate, and symptom scores of pain caused by prostate cancer.

**Ethics and dissemination:**

This systematic review will evaluate the efficacy and safety of Chinese patent medicine for pain caused by prostate cancer. Because all of the data used in this systematic review and meta-analysis has been published, this review does not require ethical approval. Furthermore, all data will be analyzed anonymously during the review process Trial.

**Trial registration number:**

PROSPERO CRD42019131544.

## 1 Introduction

Prostate cancer is a male malignant tumor disease which has a high prevalence in recent years.^[1]^ Patients who suffer from advanced prostate cancer are vulnerable to osseous metastasis which will lead to intense pain, so far as to pathological fracture, which has tremendous reduction in the quality of life.^[2]^ The secretion of prostaglandin could accelerate the bone resorption around the tumor. Due to the intense sensibility of the nerve endings, sharp pain hence generate.^[3]^ The American Cancer Society (ACS) released the data of the 2018 us Cancer research, showing that prostate cancer, lung cancer, and colon cancer have become the three major threats for American men.^[4]^ Prostate cancer led the list, accounting for more than a quarter of all new tumors. According to the latest data of the United States in 2016, the most common malignant tumors for men are prostate cancer, colorectal cancer, and melanoma.^[5]^ About 50% of patients were locally advanced when diagnosed, and about 30% had bone metastasis when diagnosed.^[6]^

In April 2015, China's tumor registration center released data reported by 234 registries nationwide, and the incidence of male prostate cancer showed an obvious growth trend.^[7]^ Endocrine therapy is always an important part of the prevention and treatment of prostate cancer. Development up to now, no matter what kind of treatment is hard to avoid the side effects of, low testosterone levels can cause such as anemia,^[8]^ cardiovascular side effects,^[9,10]^ type 2 diabetes, osteoporosis,^[11]^ and many other diseases, severe physical and psychological trauma makes it hard for some patients tolerance, give up the treatments. Modern medical philosophy not only emphasizes to improve the survival rate, but also to improve the quality of life of patients.

Cancer pain is one of the most important symptoms in patients who suffer from advanced cancer, and it greatly deteriorate the physical and psychological health, meanwhile, the quality of life of patients.^[12–14]^ The morbidity of the symptom is about 70% to 90%. According to the statistics which announced by WHO in 2003, there are about 10 million new cancer patients in the world every year, and 300 to 1000 million cancer patients fail to receive timely and effective treatment.^[15,16]^ The combination of traditional Chinese medicine and endocrine therapy for prostate cancer has become a hot spot in the field of medical research. According to the clinical manifestations of prostate cancer belongs to traditional Chinese medicine “retention of urine” “stranguria” category. Traditional Chinese medicine believes that improper diet, internal injury, external sensation of dampness, and heat are the main causes of prostate cancer.^[17]^ Chinese patent medicine is based on Chinese herbal medicine. Under the guidance of TCM theory, in order to prevent and treat diseases, it is processed into a certain dosage form of traditional Chinese medicine products according to the prescribed prescription and preparation process, which is approved by the State Drug Administration.^[18]^ A class of traditional Chinese medicine preparations. As the most important part of Traditional Chinese medicine, Chinese patent medicines have been widely used in clinical practice as derivatives of Chinese herbal medicine.^[19,20]^ However, due to the limitation of the size and number of clinical centers, most clinical trials are small samples with low-quality and lack of evidence-based exploration. Besides, the publication of the similar systematic review has not been retrieved in the database. Therefore, this review hopes to adopt meta-analysis to evaluate the efficacy and safety of Chinese patent medicine in the treatment of PE and provide evidence for its application in clinical practice.

## 2 Methods

This systematic review protocol has been registered on PROSPERO as CRD42019131544. The protocol follows the Cochrane Handbook for Systematic Reviews of Interventions and the Preferred Reporting Items for Systematic Reviews and Meta-Analysis Protocol (PRISMA-P) statement guidelines. We will describe the changes in our full review if needed.

### 2.1 Inclusion criteria for study selection

#### 2.1.1 Types of studies

Take Chinese patent medicine combined with other effective interventions as main treatment, including randomized controlled trials of the control group. Language is limited in Chinese and English. Non-randomized controlled trials, quasi-randomized controlled trials, case series, case reports, and crossover studies will be excluded.

#### 2.1.2 Types of participants

Male patients who were diagnosed with prostate cancer will be included, the type of the disease includes adenocarcinoma (adenocarcinoma), ductal adenocarcinoma, urothelial carcinoma, squamous cell carcinoma, and adenosquamous carcinoma. Meanwhile, the patients who were recruited should have the symptom of pain caused by prostate cancer, including bone pain caused by bone metastasis. However, the patients who suffer from pain caused by other factors would be excluded. Patients who suffer from cognition impairment and other severe mental illness will also be excluded. In addition, the inclusion criteria will not be restricted by region, country, nation and origin.

#### 2.1.3 Types of interventions

##### 2.1.3.1 Experimental interventions

Chinese patent medicines or the combined western medicine are used as experimental interventions. Other traditional Chinese medicine treatments such as intravenous medication, acupuncture, and massage will be limited.

##### 2.1.3.2 Control interventions

As for the control interventions, who accepted simple western medicine can be used as a control intervention or did not get any treatment as a blank control would be adopted. However, once they had accepted the therapy of TCM, the trials will be rejected.

#### 2.1.4 Types of outcome measures

##### 2.1.4.1 Primary outcomes

Adopting NRS (Numerical rating scale) of pain criteria as the main evaluation, the specific division: Painless 0 points; mild pain 1 to 3 points; moderate pain 4 to 6 points; severe pain 7 to 9 points; severe pain 10 points, 0 to 10 points representing the patient's pain level. Significantly effective: the patient's pain level is reduced by at least two or three levels, or the patient is painless; Effective: The patient's pain level is reduced by one grade or the patient presents moderate and mild pain; Invalid: The patient's pain does not have any relief, and there is even a tendency to aggravate.

##### 2.1.4.2 Secondary outcomes

1. International prostate symptom score (IPSS), the patient self-evaluation form;
2. Quality of life scores for patients with prostate cancer before and after treatment (PRSSQOL): A scale, designed by the University of California, USA, to evaluate the condition and living quality of patients who suffer from advanced, hormone-insensitive prostate cancer. It specifically includes nine aspects: Physical strength, pain, fatigue, appetite, family/marriage, constipation, mood, defecation, and overall feelings. Each item is calculated with 100 points, and the lower the score, the worse the situation.

### 2.2 Search methods for the identification of studies

We will Search PubMed, Cochrane Library, AMED, EMbase, WorldSciNet; Nature, Science online and China National Knowledge Infrastructure (CNKI), China Biology Medicine disc (CBM), China Resources Database. The temporal interval is limited from the time that the databases created to June 2019, and the combination of keyword and free word retrieval is adopted. The search terms include “Chinese patent medicine,” “proprietary Chinese medicine,” “prostate cancer,” “prostate cancer,” “pain,” “Random Control.” The complete PubMed search strategy is shown in Table 1.

**Table 1:**
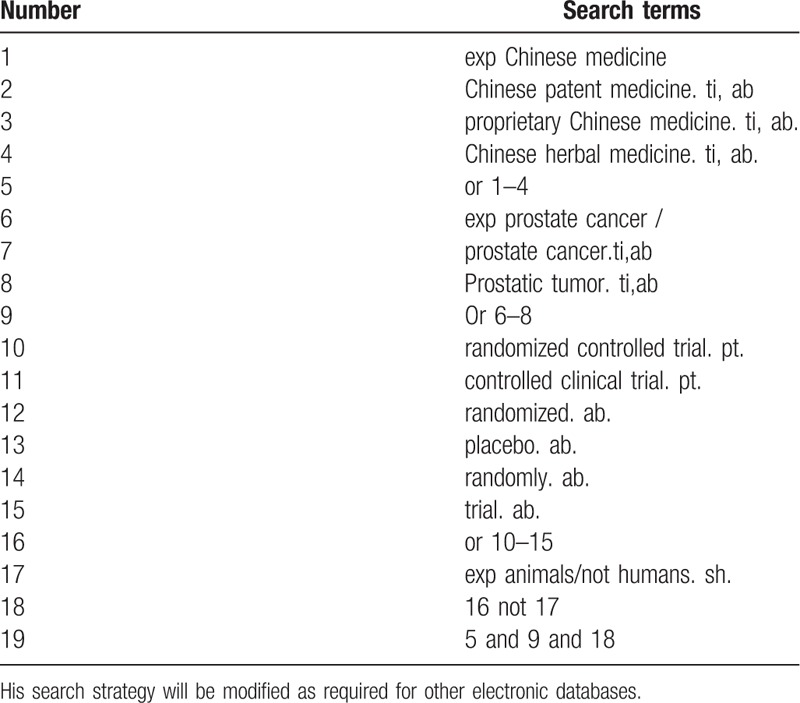
Search strategy used in PubMed database.

### 2.3 Data collection and analysis

#### 2.3.1 Selection of studies

Initial evaluations were performed independently by two investigators through filtrating the titles and abstracts of each documents in the Endnote database, eliminating duplicates and documents that were clearly inconsistent with the study. After the preliminary assessment, in order to screen out eligible trials, the full text of the selected literature would be evaluated, which mainly aims at whether there were problems just like uncontrolled studies, no randomization, inconsistent assessment criteria, and similar data. When the two researchers could not reach a consensus, the third judge would make the final judgment.

#### 2.3.2 Data extraction and management

There will be two investigators independently extract information from the included literature, mainly contents: Author (year), sample size, disease stage, patient age, intervention factors, control factors, intervention time, observation index, NRS score, symptoms Ratings, quality of life scores, etc. The extracted literature data is filled in a unified data statistics table.

#### 2.3.3 Assessment of risk of bias in included studies

Two investigators independently assess the quality of the included literature by using the Cochrane Collaboration's bias risk assessment tool. The assessment includes: random sequence generation, allocation concealment, blinding, incomplete outcome data, selective outcome reporting, and other possible biases. According to the relevant standards in the *Cochrane Intervention System Evaluation Manual*, it is divided into low risk, high risk, and unclear.

#### 2.3.4 Dealing with missing data

In the event of data loss during the screening and extraction of literature data, primarily, we would actively look for the cause of the loss, and then we would contact the experimental research author by telephone, mail, etc. to retrieve the lost data. If the loss could not be retrieved, we will only extract and analyze the useful data, and the situation would be indicated.

#### 2.3.5 Data synthesis and analysis

**Figure 1:**
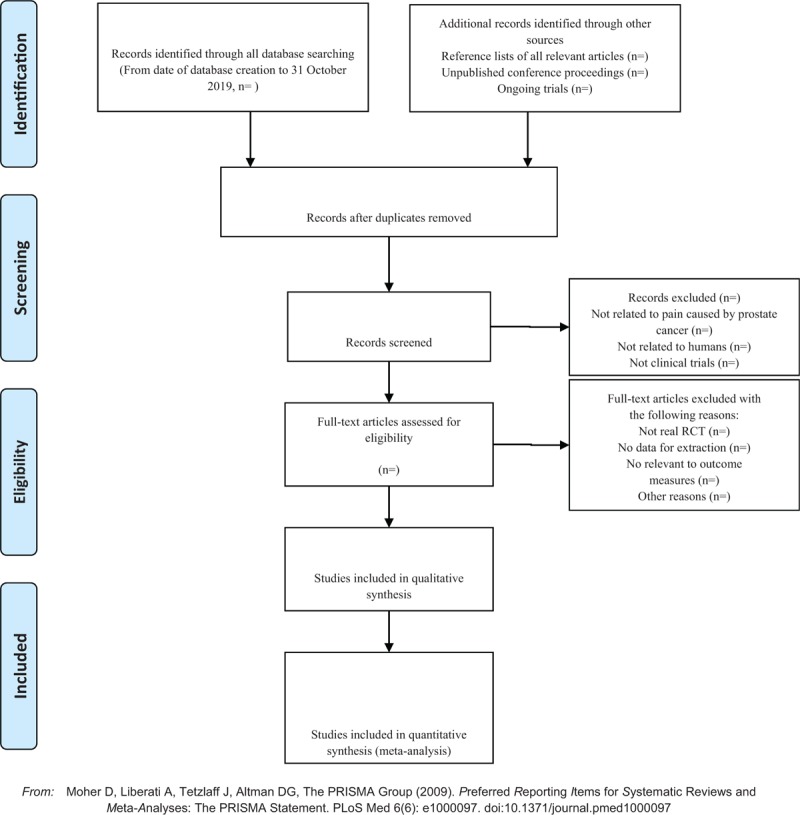
The PRISMA flow chart.

The analysis of the data will adopt RevMan 5.3 software. As for the two categorical variables, we select relative risk (RR) or odds ratio (OR) and 95% CI. As for the continuous variables, we select weighted mean difference (WMD) or standard mean difference (SMD) and 95% CI, the difference would be statistically significant when *P* < .05. Heterogeneity test would be analyzed by using chi-square test. When *P* ≥ .1, the difference was considered to be not statistically significant. When *P* < .1, *I*^2^ > 50%, the random effect model would be used, as for the other situation, the fixed effect model would be adopted. For studies that provide baseline and post-treatment data, we will estimate the change values by the method recommended by Cochrane. The details of selection process will be shown in the PRISMA flow chart (Fig. 1).

#### 2.3.6 Assessment of heterogeneity

If there is significant heterogeneity between a group of studies, we will explore the reasons for the existence of heterogeneity from various aspects, such as the characteristics of the subjects and the degree of variation of the interventions. Necessarily, sensitivity analysis or subgroup analysis would be adopted to explain the heterogeneity.

#### 2.3.7 Assessment of publication bias

The forest map and funnel plot would be drawn and analyzed by Rev Man5.3 software, and the funnel plot would be used to analyse the potential publication bias.

#### 2.3.8 Grading the quality of evidence

The quality of evidence for the main outcomes will also be assessed with the GRADE approach. The evaluation included bias risk; heterogeneity; indirectness; imprecision; publication bias. And each level of evidence will be made “very low,” “low,” erate,” or “high” judgment.

## 3 Discussion

At present, pain has been the fifth vital sign, which together with respiration, blood pressure, pulse, and body temperature. In 1996, the American Pain Association (APA) first proposed the concept of pain and its control, which indicated that an effective pain relief program for patients is a basic requirement for clinical medical work. It has been a difficult problem to the treatment of pain caused by prostate cancer and its bone metastasis in the middle and advantaged stage, which has belonged to refractory cancer pain.^[21,22]^ Recent years have witnessed an increase on the study of treating cancer pain, vast researches have indicated that analgesics have great efficiency in ameliorating pain, but the pesticide effects are short, and there are kinds of adverse reaction, such as kidney damage, liver damage, cardiovascular and cerebrovascular damage, digestive disease, etc.^[23,24]^ Generally speaking, numerous patients who suffer from cancer could not gain satisfied control effect. Moreover, these patients need long-term use of analgesic drugs to ameliorate pain, which also directly increase the cost of therapy for patients.

Traditional Chinese medicine, has been proved that it is a safe, feasible, and effective treatment. The clinical operation and theory of TCM have been inherited and carried forward by the majority of clinicians, especially in the treatment of pain. This therapy could ameliorate vast kinds of pain efficiently, and there is no adverse reaction which could insure the security.^[25]^ Chinese patent medicine is based on traditional Chinese medicine. Under the guidance of traditional Chinese medicine theory, in order to prevent and treat diseases, it is processed into a certain dosage form of traditional Chinese medicine products according to the prescribed prescription and preparation process, which is approved by the State Drug Administration. A class of traditional Chinese medicine preparations. As the most important part of Traditional Chinese medicine, Chinese patent medicines have been widely used in clinical practice as derivatives of Chinese herbal medicine and the recent studies have shown that the therapy could ameliorate the pain cause by prostate cancer to some extent and improve the quality of the life.^[26]^

As far as we know, there has not made any comparison of the effectiveness of Chinese patent medicines in the treatment of prostate cancer pain. Therefore, we will use systematic review and meta-analysis to evaluate the efficacy and safety of Chinese patent medicines for the treatment of pain caused by prostate cancer. We expect that the review could provide a basis for Chinese patent medicines treatment of pain caused by prostate cancer and offer more and better options for the treatment to patients.

## Author contributions

**Conceptualization:** Hong Li.

**Data curation:** Xu-dong Yu.

**Funding acquisition:** Xiaoyong Gong, Xu-dong Yu.

**Methodology:** Xiaoyong Gong, Ji-sheng Wang.

**Project administration:** Yuan-yuan Li.

**Resources:** Ji-sheng Wang, Yuan-yuan Li, Yi Lei.

**Software:** Xu-dong Yu, Rui-jia Liu, Li-yuan Chu, Yi Lei.

**Supervision:** Rui-jia Liu.

**Validation:** Rui-jia Liu, Hong Li.

**Visualization:** Hong Li.

**Writing – original draft:** Li-yuan Chu.
